# Evaluating the Effect of Drug Provocation Tests on Anxiety and Hopelessness Levels

**DOI:** 10.3390/medicina61061014

**Published:** 2025-05-29

**Authors:** Gürgün Tuğçe Vural Solak, Kurtuluş Aksu, Melis Yağdıran, Fatma Dindar Çelik, Özgür Akkale, Onur Telli, Hatice Çelik Tuğlu, Nur Betül Baştuğ İnan, Özge Göktürk, Yavuz Karahan, Yavuzalp Solak

**Affiliations:** 1 Division of Immunology and Allergy, Ankara Ataturk Sanatoryum Training and Research Hospital, University of Health Sciences, Ankara 06290, Türkiye; kurtulusaksu@yahoo.com (K.A.); mlstemizkan@hotmail.com (M.Y.); fatmadindar@gmail.com (F.D.Ç.); akkale26@gmail.com (Ö.A.); onrtlli@gmail.com (O.T.); haticecelik830@gmail.com (H.Ç.T.); bastugbetul@gmail.com (N.B.B.İ.); gktrkgs@hotmail.com (Ö.G.); yavuzkarahan1987@gmail.com (Y.K.); 2 Çubuk District Health Directorate, Ankara 06760, Türkiye; yavuzalp80@gmail.com

**Keywords:** drug provocation test, anxiety, hopelessness

## Abstract

*Background and Objectives:* Drug provocation tests (DPTs) diagnose drug hypersensitivity reactions (DHRs) and identify safe alternatives. DHRs contribute to patient anxiety. The aim of this study was to evaluate anxiety and hopelessness levels before and after DPT and to examine patients’ avoidance characteristics of the tested drugs. *Materials and Methods:* Patients undergoing DPT were included. The State–Trait Anxiety Inventory (STAI) assessed anxiety, and the Beck Hopelessness Scale measured hopelessness. Demographic and clinical data, pre- and post-DPT scores, the status of using the medication provided after DPT, and the reason for not using it were analyzed. *Results:* Seventy-nine patients (60 female, 75.9%) participated. Patients’ Beck Hopelessness Scale, STAI–State, and STAI–Trait scores decreased significantly after the DPT compared to the initial scores. Among the patients who developed a reaction during the drug provocation test, the rate of those whose scale scores increased was significantly higher than the rate of those who did not develop a reaction. A total of 42 patients (53.2%) did not use the alternative safe drug. Of these, six (14.3%) reported that their reluctance stemmed from a fear of experiencing a reaction similar to their initial adverse event. Patients with concomitant allergic diseases were less likely to use alternative safe drugs. *Conclusions:* DPT reduces long-term anxiety and hopelessness. However, one in seven patients avoids the prescribed safe drug due to fear of recurrence. Effective communication, especially with patients who have allergic conditions or experience a reaction during DPT, and psychological support may improve adherence to the tested medication.

## 1. Introduction

The gold standard for confirming drug hypersensitivity reactions (DHRs) is the drug provocation test (DPT) [1,2]. Beyond diagnostic confirmation, DPTs serve various other clinical purposes. These include identifying a safe alternative drug, ruling out hypersensitivity in cases where clinical symptoms do not clearly indicate a drug allergy, and assessing the risk of cross-reactivity when medications from the same pharmacological class are required [1].

A study comparing patients with allergic diseases to healthy volunteers found that individuals with allergies are more vulnerable to psychopathological conditions, including loneliness, stress, obsession, and depression. These findings suggest that allergic patients experience compromised psychological well-being [3]. Consistent with other allergic conditions, patients with DHRs have been reported to have higher levels of anxiety [4,5]. The psychological burden associated with DHRs is likely due to the wide range of clinical manifestations, from skin-related symptoms to severe outcomes such as death. Given the potential for recurrence upon re-exposure to the drug, the heightened anxiety levels in this patient group are understandable. A study on psychiatric disorders in patients with asthma, allergic rhinitis, and drug hypersensitivity found that psychiatric comorbidities were most prevalent in the drug hypersensitivity group [6].

Hopelessness, defined as the perception of having no control over future events and harboring negative expectations about the future, has been strongly associated with depression and the development and progression of suicidal tendencies [7,8]. However, no data on the levels of hopelessness in patients with drug hypersensitivity reactions have been identified in the literature.

Despite the available evidence, data on the mental health status of patients with a history of drug hypersensitivity reactions are limited [9,10]. However, to the best of our knowledge, no studies have evaluated the long-term anxiety and hopelessness levels of patients after DPT, as addressed in our study. This study holds significance, as it provides the first data in the literature on this topic.

This study primarily aimed to assess state and trait anxiety and hopelessness levels before and long after DPT in patients with a history of drug hypersensitivity who underwent testing to identify a safe alternative. Additionally, we examined their use of these alternatives post-DPT. We hypothesized that anxiety and hopelessness would be high in patients who avoided the alternative despite a negative DPT. The secondary objective was to identify characteristics of those who refrained from using the prescribed safe alternatives.

## 2. Materials and Methods

### 2.1. Study Design and Study Population

This descriptive and prospective longitudinal (two time points) study was conducted between March and August 2024. It included patients with a reliable history (clinician assessment based on detailed history and previous documentation) of DHR who presented to a tertiary allergy clinic. The inclusion criteria were: (1) being 18 years of age or older, (2) undergoing a drug provocation test for alternative safe drug identification, and (3) voluntarily agreeing to participate in the study. Exclusion criteria included: (1) being under 18 years of age, and (2) declining to participate. The sample size was calculated using G*Power version 3.1.9.2. [11]. Considering that before-and-after comparisons would be made with the Wilcoxon test, a one-way hypothesis was established with α = 0.05 and β = 0.80 for low effect size, and sample size was calculated. The calculated sample size was determined as 74. In order to increase the reliability, it was decided that the number of participants should be 10% more than the target number and the minimum number of patients involved in the study should be 82. Three patients with missing data were excluded from the analysis.

### 2.2. Demographic and Clinical Characteristics of Patients

The clinician recorded demographic and clinical characteristics on the Case Record Form, including age, gender, education, occupation, comorbid allergic and other diseases, suspected drug group, initial reaction symptoms and severity, time between the initial reaction and drug provocation test (<6 or ≥6 months), occurrence and severity of reactions during the DPT, and post-DPT drug use. Immediate type reactions during both the initial reaction and provocation test were classified per the World Allergy Organization’s 2019 update [12].

### 2.3. Classification of Drug Hypersensitivity Reactions

DHRs were classified into immediate and non-immediate types. Reactions occurring within 1 to 6 h were classified as immediate type reactions, whereas reactions such as maculopapular eruptions, organ involvements, etc., occurring after 6 h were classified as non-immediate type reactions [12,13]. Drug hypersensitivity reactions were further evaluated for the presence of anaphylaxis according to the European Academy of Allergy and Clinical Immunology 2021 guideline [14].

### 2.4. Administration of Drug Provocation Tests and Management of Patients

The protocols used for DPT followed internationally accepted guidelines [12,15]. The clinician evaluated each patient’s medical history, comorbidities, and DHR before testing. All DPTs were supervised by an experienced physician and nurse in a clinical setting equipped for emergencies.

A placebo was administered before each test, but placebo-induced reactions were excluded from the analysis. The DPT was conducted the next day in divided doses, with continuous monitoring of vital signs and clinical findings. Patients were observed for two hours after the final dose and scheduled for a follow-up. In subsequent patient visits, patients were questioned for non-immediate reactions. A positive DPT result was defined by the presence of any objectively documented sign or symptom [1,2]. Patients with negative results were informed that the identified alternative drug was safe for use if needed.

### 2.5. Assessment of Anxiety Level

The State–Trait Anxiety Inventory (STAI) was used to assess anxiety severity through self-report. Developed by Spielberger et al., its Turkish validity and reliability have been established [16,17]. This inventory is designed to evaluate both the baseline anxiety level of individuals as a trait and the changes in anxiety triggered by specific external stimuli. It consists of two subscales: STAI–State (STAI-S) and STAI–Trait (STAI-T). The STAI-S measures the anxiety felt by the individual “at this moment” under certain conditions, while the STAI-T assesses the individual’s general anxiety level. Each subscale includes 20 questions, for a total of 40 questions. The STAI-S uses a Likert-type scale. The minimum possible score on each scale is 20 points, and the maximum possible score is 80 points. A higher score on either scale indicates a higher level of anxiety.

### 2.6. Assessment of Hopelessness Level

The Beck Hopelessness Scale was utilized to assess the patients’ negative perspectives regarding the future, specifically their level of hopelessness [18]. The Turkish validity and reliability of the scale were established by Seber et al. in 1993 [19]. The scale consists of 20 items, each with two response options: “yes” or “no”. A higher score on the scale indicates a higher level of hopelessness. The scale has a minimum possible score of 0 and a maximum score of 20.

Follow-up evaluations (STAI and Beck Hopelessness Scale) were assessed 1 month after patients underwent DPT. If the baseline scale scores were higher than the follow-up scale scores, it was called the “Decreased Group”, and if the baseline scale scores were lower than the follow-up scale scores, it was called the “Increased Group”. Their usage of the safe alternative drugs identified in the test was queried. Additionally, if the patient did not use the safe alternative drug, the reasons for this decision were investigated.

### 2.7. Statistical Analysis

Statistical analyses were performed using IBM SPSS Statistics for Windows, Version 25.0 (IBM Corp., Armonk, NY, USA). Descriptive statistics were presented as frequency, percentage, mean, median, standard deviation, and minimum–maximum values. Pearson’s Chi-squared test was used for categorical data analysis, and Fisher’s Exact Test was applied for values less than five in 2 × 2 contingency tables. The Kolmogorov–Smirnov test assessed the normality of data distribution. For quantitative data in independent groups, the Student’s *t*-test was used for normally distributed data, while the Mann–Whitney U test was applied for non-normally distributed data. The Wilcoxon test compared scale scores before and after the intervention. Normally distributed variables were expressed as mean ± standard deviation, and non-normally distributed variables as median with minimum and maximum values. Independent variables included demographic data, comorbid allergic diseases, other comorbidities, the grade of the initial reaction, presence of anaphylaxis during the initial reaction, immediate vs. non-immediate reactions, and reaction development during the drug provocation test. Dependent variables were Beck Hopelessness Scale, STAI-S, and STAI-T scores. Statistical significance was set at *p* < 0.05. The effect size calculations were performed using Python v3.12.7 with the support of the pandas, numpy, and statsmodels libraries. In the post hoc power analysis, BECK scale, STAI-S and STAI-T were evaluated and found to be 0.81, 0.974 and 0.992, respectively.

## 3. Results

### 3.1. Demographic and Clinical Characteristics of the Patients

A total of 79 patients, with a mean age of 43.61 ± 11.39 years, including 60 women (75.9%), were enrolled in the study. The most common drugs suspected of causing hypersensitivity reactions were nonsteroidal anti-inflammatory drugs (NSAIDs) and beta-lactam antibiotics, accounting for 53.2% and 50.6%, respectively. Wheal and angioedema were the most commonly observed manifestations during the initial hypersensitivity reaction (79.2% and 62%, respectively). Other demographic and clinical characteristics of the patients are presented in Table 1.

### 3.2. Evaluation of Drug Provocation Test Results

All patients underwent DPT to identify an alternative safe drug. Fifteen patients (19%) developed reactions during drug provocation testing. Other features associated with DPT are presented in Table 2.

### 3.3. Assessment of Anxiety and Hopelessness Level

The patients’ Beck scores were higher before DPT (median: 4; range 1–15) than after DPT (median: 3, IQR: 0–18) (*p* = 0.000, Z = −4.014). The patients’ STAI-S scores were higher before DPT (median: 37; range: 19–70) than after DPT (median: 30, range: 16–65) (*p* = 0.000, Z = −5.511). The patients’ STAI-T scores were higher before DPT (median: 43; range: 26–63) than after DPT (median: 36, range: 20–66) (*p* = 0.000, Z = −6.157) (Table 3). A total of 12 patients (15.2%) had increased Beck, STAI-S and STAI-T scores, while 67 patients (84.8%) had decreased scores (Table 4).

### 3.4. Evaluation of the Changes in Beck Hopelessness Scale, STAI-S and STAI-T Scores

There was no statistical difference between the groups whose Beck Hopelessness Scale, STAI-S and STAI-T scores decreased and increased after 1 month in terms of age, gender, educational status, occupation, comorbidities, grade of initial reaction, presence of anaphylaxis history, and type of initial reaction (respectively, *p* = 0.442, *p* = 0.516, *p* = 0.507, *p* = 0.186, *p* = 0.562, *p* = 0.618, *p* = 0.877, *p* = 0.241). Among the patients who developed a reaction during the drug provocation test, the rate of those whose scale scores increased (40.0%) was significantly higher than the rate of those who did not develop a reaction (9.4%) (*p* = 0.003, x^2^ = 8.847) (Table 4).

### 3.5. Evaluation of Patients’ Use of Alternative Safe Medicines

Among the participants, 37 patients (46.8%) reported using the alternative safe medication identified following DPT. These patients were categorized as Group A. In contrast, 42 patients (53.2%) did not use the alternative safe medication. Of these, six patients (14.3%) cited the fear of experiencing reactions similar to the initial side effects as the reason for non-use, and these patients were classified as Group B. The remaining 36 patients (85.7%) indicated that they did not use the medication due to the lack of a clinical indication (Table 5).

While 26.3% (*n* = 5) of 19 patients with allergic diseases refrained from using the medication, despite needing it, due to fear, this rate was significantly lower in the group without an allergic disease (4.2%, *n* = 1) among 24 patients (*p* = 0.037, x^2^ = 4.333). The median Beck Hopelessness Scale scores measured after DPT were 3.0 (range: 0–16) in Group A and 6.0 (range: 2–17) in Group B; this difference was statistically significant (*p* = 0.010, Z = −2.561). The post-DPT STAI-T scores for Group A were significantly lower than those for Group B (*p* = 0.031, *t* = −2.750). No statistically significant differences were observed between Group A and Group B in terms of age, gender, education level, occupation, comorbidities, grade of initial reaction, presence of anaphylaxis, type of initial reaction, presence of a reaction during DPT, or other scale scores (Table 5).

## 4. Discussion

Our study is, to the best of our knowledge, the first in the literature to evaluate the long-term state/trait anxiety and hopelessness levels and related factors in adult patients with a reliable history of drug hypersensitivity reactions undergoing DPT. In this study, patients’ Beck Hopelessness Scale, STAI-S, and STAI-T scores decreased significantly after the drug provocation test compared to the initial scores. The rate of patients who developed reactions during DPT and showed an increase in state/trait anxiety and hopelessness scores was significantly higher than the rate of those who did not develop reactions. Patients with concomitant allergic diseases were less likely to use alternative safe drugs.

Beken et al. evaluated the anxiety levels of mothers of children with suspected food allergies. Mothers completed the STAI before and a few months after the Oral Food Challenge (OFC). Compared to the healthy control group, the STAI-S and STAI-T scores of mothers were statistically higher at baseline in cases of suspected food allergy in their children, indicating that the mothers experienced elevated anxiety due to the suspected food allergy. After re-evaluation a few months after OFC, it was observed that the mothers’ STAI-S scores, and consequently their anxiety levels, decreased after the OFC, regardless of whether the results were negative or positive. However, the decrease was more pronounced in the group with negative OFC results [20]. Similar to food allergies, the presence of drug allergies is also associated with high anxiety and depression scores, and DPT-related anxiety has also been demonstrated in patients with drug allergies [5,9,21]. In our study, similar to the aforementioned studies, we found that the Beck Hopelessness Scale, STAI-S, and STAI-T scores of patients significantly decreased after the drug provocation test in the long term. The unique aspect of our study is its evaluation of anxiety and hopelessness scores after a long-term period. It is crucial to evaluate this patient group with a multidisciplinary approach and conduct detailed allergic and psychological assessments to improve their quality of life. Furthermore, we believe that patients with reduced anxiety and hopelessness levels are more likely to show higher treatment compliance.

Another significant finding in our study was that the proportion of patients who exhibited an increase in both state/trait anxiety and hopelessness scores during DPT was notably higher compared to those who did not develop a reaction. This suggests that experiencing a reaction during DPT may serve as an obstacle to alleviating anxiety and hopelessness levels. This finding aligns with the study conducted, where it was observed that the STAI-S scores of mothers undergoing OFC for their children decreased a few months after the test, regardless of whether the results were negative or positive, with a more pronounced decrease observed in the negative OFC result group [20]. It is well established that patients with a history of drug hypersensitivity reactions often harbor fears of experiencing similar reactions again [22,23,24]. This could account for the higher anxiety and hopelessness scores seen in patients who reacted during DPT. We recommend that physician–patient communication be prioritized and that patients are thoroughly informed about the distinct nature of each drug provocation test, which involves different medications.

Asthma, along with conditions such as rhinitis, is often associated with increased loneliness, stress, obsession, variability, anxiety, and the desire to escape separation, contributing to its exacerbation [3,4]. This situation may lead to chronicity, resulting in emotional deficits and deprivation, which can also impair treatment adherence. However, the continuity of medication behaviors is not consistent, and patients typically do not experience this cycle repeatedly during the course of medication use. Despite this, patients with drug hypersensitivity reactions, who face separation and the associated fears, often experience a diminished quality of life [5]. Patients with analgesic intolerance and cyclooxygenase-2 inhibitors often undergo long-term care evaluation, and while there is improvement after negative drug provocation tests, fear of experiencing similar reactions during the tests remains a significant barrier to using the safe alternatives [22]. Despite this, psychological evaluations concerning medication use and adherence have been explored in these patients to a limited extent. In our study, among 79 patients who were provided with alternative medications after a successful DPT, 37 patients (46.8%) (Group A) used the tested medication. Six patients (14.3%) (Group B), however, did not use the alternative medication despite the indication, due to the fear of experiencing a reaction similar to the initial one. Since Group B included relatively few patients, the data obtained from the study should be evaluated with caution. It should be noted that the results indicate a possible relationship. Among the reasons why patients do not use the safe alternative drug provided, the fact that 85.7% (n = 36) of them do not need the medicine is an important and frequently encountered situation in our daily practice. Similarly, Soyyiğit et al. reported that 15.9% of patients did not use safe alternative medications despite the indication, citing the risk of recurrence of previous drug reactions as the primary reason [9]. These findings may suggest insights into the importance of patient education to help individuals manage their condition and improve outcomes.

When examining the characteristics of the patients in our study, those with concomitant allergic diseases were less likely to use the alternative safe medications. The presence of additional allergic diseases has been associated with higher levels of anxiety, which may explain the avoidance of drug use in patients with elevated anxiety levels. In support of this, we found that the pre-DPT STAI-T scores, reflecting the general anxiety levels of patients who did not use the drug despite the indication (Group B), were higher than those of patients who used the drug (Group A), although the difference was not statistically significant (*p* = 0.216). Bommarito et al. also compared patients who used and did not use NSAID medications deemed safe after DPT and found that the group who did not use the medication was statistically older [24]. In our study, Group B had a higher mean age compared to Group A, although the difference was not statistically significant (*p* = 0.284). This age difference may be linked to the presence of comorbid diseases and the tendency for older patients to avoid taking risks. As age increases, patients may become more cautious about using medications, even if they are deemed safe, due to a fear of experiencing a recurrence of adverse reactions. For this reason, it is crucial to establish effective communication with older patients and those with concomitant allergic diseases, reassuring them that they can safely use a drug that has tested negative in a DPT.

When patients were re-evaluated, those who did not use the safe alternative drug showed a more significant increase in their Beck Hopelessness Score and STAI-T scores. Although STAI-S scores were higher in Group B, the difference was not statistically significant. These results support the hypothesis of our study. This situation may suggest that patients with increased anxiety and hopelessness levels do not use the medication because they are truly afraid, and it can also be explained by the fact that the person’s anxiety and hopelessness scores may have increased even more due to not being able to use the safe alternative medication provided and feeling insecure. However, it should be kept in mind that the number of this patient group is particularly low and the results should be evaluated with caution. Larger-cohort studies are needed for definitive results.

A limitation of our study is the inability to evaluate individual factors such as physical and mental health issues and confounding factors that may have affected anxiety and hopelessness levels in the patient group whose scores were assessed after a long period. In our study, the drug classes responsible for hypersensitivity reactions, as well as the drug classes and the number of drugs tested through drug provocation, were not evaluated, as these were not among the objectives of the study. Although we questioned the patients, the 2 h observation period may have caused the immediate reactions that occurred later to be missed. Since the number of patients who stated that they did not use a safe alternative drug due to fear of experiencing a recurrence was very low, no definite relationship could be shown in comparisons where this patient group was included. However, the existence of a possible relationship may guide larger cohort studies.

## 5. Conclusions

DPT reduced long-term anxiety and hopelessness in patients with a reliable history of drug hypersensitivity. The clinical importance of our study is that it raises awareness among physicians about the need to evaluate psychological states before and after DPT and to provide support if necessary. However, due to the limited number of patients, more comprehensive and prospective studies are needed to better understand the complex relationship between DPT and psychological states.

## Figures and Tables

**Table 1:** Demographic and clinical characteristics of the patients.

Characteristics Values	
Female/Male, *n* (%)	60 (75.9)/19 (24.1)
Age (y), mean	43.61 ± 11.39
Female	44.2 ± 10.8
Male	41.5 ± 13.1
Education level, *n* (%)	
Primary school	20 (25.3)
Secondary school	10 (12.7)
High school	18 (22.8)
University	31 (39.2)
Occupation, *n* (%)	
Not employed	34 (43)
Employed	38 (48.1)
Retired	5 (6.3)
Student	2 (2.5)
Concomitant allergic disease, *n* (%)	34 (43)
Allergic rhinitis	8 (10.1)
Asthma	26 (32.9)
Chronic urticaria	10 (12.6)
Venom hypersensitivity	1 (1.2)
Food hypersensitivty	2 (2.5)
Nasal polyp	1 (1.2)
Concomitant other diseases, *n* (%)	40 (50.6)
Psychiatric disease	4 (5.1)
Suspected culprit drug, *n* (%)	
Antibiotics	50 (62.5)
Beta lactam	40 (50.6)
Quinolone	7 (8.9)
Macrolide	13 (16.5)
Other antibiotics	11 (13.9)
NSAIDs,	42 (53.2)
Other drugs	44 (55.7)
Duration since the last drug hypersensitivity reaction, *n*	
<6 months	37 (46.8)
≥6 months	42 (53.2)
Type of symptoms of the initial drug hypersensitivity reactions, *n* (%)	
Wheal	63 (79.7)
Angioedema	49 (62)
Dyspnea	23 (29.1)
Anaphylaxis	12 (15.2)
Gastrointestinal symptoms	16 (20.3)
Cardiovascular symptoms	10 (12.7)
Neurological symptoms	19 (24.1)
Maculopapular exanthema	7 (8.9)
Other symptoms	9
Grade of initial drug hypersensitivty reactions, *n* (%)	72 (91.1)
Grade 1	30 (38)
Grade 2	4 (5.1)
Grade 3	27 (34.2)
Grade 4	1 (1.3)
Grade 5	14 (17.7)

Abbreviations: NSAID, nonsteroidal anti-inflammatory drug.

**Table 2:** Features of drug provocation tests.

Variables Values	
Drugs	
Antibiotics	54 (68.3)
NSAID	42 (53.2)
Local anesthetic drugs	9 (11.4)
PPI	2 (2.5)
Other drugs	22 (27.8)
Presence of reaction that developed during DPT, *n* (%)	
Yes	15 (19)
No	64 (81)
Reactions that developed during a positive DPT, *n* (%)	
Pruritus	3 (3.7)
Erythema	4 (5)
Wheal	9 (11.3)
Angioedema	4 (5)
Nause	1 (1.3)
Nasal congestion	1 (1.3)
Headache	1 (1.3)
Stomachache	1 (1.3)
Hypotension	1 (1.3)
Maculopapular eruption	4 (5)
Grade of reactions that developed during a positive DPT, *n* (%)	
Grade 1	10 (66)
Grade 2	2 (13.3)
Grade 3	1 (6.6)
Grade 4	None
Grade 5	1 (6.6)

Abbreviations: NSAID, nonsteroidal anti-inflammatory drug; PPI, proton pump inhibitors; DPT, drug provocation test.

**Table 3:** Beck Hopelessness Scale, STAI-S and STAI-T values of patients before and after drug provocation test.

	n	Min.	Max.	25th	50th (Median)	75th	Z *	*p*	Effect Size
Beck Scale Before DPT	79	1.00	15.0	2.0	4.0	6.0	−4.014	0.000	0.452
After DPT	79	0.0	18.0	1.0	3.0	4.0			
STAI-S Before DPT	79	19	70	27.0	37.0	44.0	−5.511	0.000	0.62
After DPT	79	16	65	24.0	30.0	37.0			
STAI-T Before DPT	79	26	63	37.0	43.0	49.0	−6.157	0.000	0.693
After DPT	79	20	66	28.0	36.0	42.0			

* Wilcoxon test was used. Abbreviations: STAI-S, State–Trait Anxiety Inventory-State Subscale; STAI-T, State–Trait Anxiety Inventory-Trait Subscale; DPT, drug provocation test.

**Table 4:** Characteristics of patients with increasing and decreasing scale scores.

	*n*	Scale Score		Test Value *p*-Value	
		Decreased Group (*n* = 67)	Increased Group (*n* = 12)		Effect Size
Age	79	43.24 ± 11.72	45.67 ± 9.48	−0.786 ^Ω^ 0.442	0.246
Gender Female Male	60 19	50 (83.3%) 17 (89.5%)	10 (16.7%) 2 (10.5%)	0.422 ^¥^ 0.516	0.073
Education level Primary and secondary school High school and university	30 49	25 (83.3%) 42 (85.7%)	5 (16.7%) 7 (14.3%)	- 0.507 ^¶^	-
Occupation Employed and student Retired and not employed	40 39	32 (80.0%) 35 (89.7%)	8 (20.0%) 4 (10.3%)	- 0.186 ^¶^	-
Concominant allergic disease Yes No	34 45	27 (79.4%) 40 (88.9%)	7 (20.6%) 5 (11.1%)	1.350 ^¥^ 0.245	0.131
Concomitant other disease Yes No	40 39	33 (82.5%) 34 (87.2%)	7 (17.5%) 5 (12.8%)	0.336 ^¥^ 0.562	0.065
Concomitant psychiatric disease Yes No	4 75	3 (75%) 64 (85.3%)	1 (25%) 11 (14.7%)	0.315 ^¥^ 0.575	0.063
Grade of initial immediate reaction, median (range)	79	3 (1–5) 2.5 ± 1.4	3 (1–5) 2.7 ± 1.6	−0.499 ^§^ 0.618	0.056
Presence of anaphylaxis Yes No	12 67	10 (83.3%) 57 (85.1%)	2 (16.7%) 10 (14.9%)	0.024 ^¥^ 0.877	0.017
Type of initial reaction Non-immediate Immediate	7 72	7 (100%) 60 (83.3%)	0 (0%) 12 (16.7%)	1.376 ^¥^ 0.241	0.132
Presence of reaction that developed during DPT Yes No	15 64	9 (60.0%) 58 (90.6%)	6 (40.0%) 6 (9.4%)	8.847 ^¥^ 0.003	0.335

Abbreviations: STAI-S, State-Trait Anxiety Inventory-State Subscale; STAI-T, State-Trait Anxiety Inventory-Trait Subscale; DPT, drug provocation test. ^Ω^ Student-*t* test values are shown as mean ± standard deviation. ^¥^ Chi-square test was used. ^¶^ Fisher’s Exact Test was used. ^§^ Mann Whitney-U test values are shown as median (min–max).

**Table 5:** Characteristics of patients according to their use of safe alternative drugs.

	n	Safe Drug Use Status		Test Value *p*-Value	
		Took Alternative Drug (*n* = 37) Group A	Did Not Take Alternative Drug (*n* = 6) Group B		Effect Size
Age	43	42.49 ± 11.36	47.50 ± 9.60	−1.154 ^Ω^ 0.284	0.508
Gender Female Male	30 13	25 (83.3%) 12 (92.3%)	5 (16.7%) 1 (7.7%)	0.608 ^¥^ 0.435	0.119
Education level Primary and secondary school High school and university	21 22	17 (81.0%) 20 (90.9%)	4 (19.0%) 2 (9.1%)	- 0.309 ^¶^	-
Occupation Employed and student Retired and not employed	20 23	17 (85.0%) 20 (87.0%)	3 (15.0%) 3 (13.0%)	- 0.597 ^¶^	-
Concomitant allergic disease Yes No	19 24	14 (73.7%) 23 (95.8%)	5 (26.3%) 1 (4.2%)	4.333 ^¥^ 0.037	0.317
Concomitant other disease Yes No	20 23	17 (85.0%) 20 (87.0%)	3 (15.0%) 3 (13.0%)	0.034 ^¥^ 0.853	0.028
Concomitant psychiatric disease Yes No	1 42	1 (%100) 36 (85.7%)	0 (%0) 6 (14.3%)	0.166 ^¥^ 0.684	0.062
Grade of initial reactions, median (range)	43	3 (1–5)	2 (1–3)	−1.005 ^§^ 0.315	0.153
Presence of anaphylaxis Yes No	5 38	4 (80.0%) 33 (86.8%)	1 (20.0%) 5 (13.2%)	0.172 ^¥^ 0.678	0.063
Type of initial reaction Immediate Non-immediate	38 5	5 (%100) 32 (84.2%)	0 (%0) 6 (15.8%)	0.917 ^¥^ 0.338	0.146
Presence of reaction that developed during DPT Yes No	8 35	6 (75.0%) 31 (88.6%)	2 (25.0%) 4 (11.4%)	0.999 ^¥^ 0.318	0.152
1st Beck scale score	43	4 (1–11)	4 (2–14)	−0.907 ^§^ 0.365	0.138
2nd Beck scale score	43	3 (0–16)	6 (2–17)	−2.561 ^§^ 0.01	0.391
1st STAI-S score	43	35.59 ± 10.12	34.67 ± 12.20	0.177 ^Ω^ 0.865	0.078
1st STAI-T score	43	41.89 ± 8.14	48.0 ± 10.31	−1.382 ^Ω^ 0.216	0.608
2nd STAI-S score	43	28.73 ± 8.88	37.67 ± 10.40	−1.990 ^Ω^ 0.092	0.876
2nd STAI-T score	43	34.43 ± 9.59	47.17 ± 10.66	−2.750 ^Ω^ 0.031	1.21

Abbreviations: STAI-S, State–Trait Anxiety Inventory-State Subscale; STAI-T, State–Trait Anxiety Inventory-Trait Subscale; DPT, drug provocation test. ^Ω^ Student-*t* test values are shown as mean ± standard deviation. ^¥^ Chi-square test was used. ^¶^ Fisher’s Exact Test was used. ^§^ Mann Whitney-U test values are shown as median (min–max).

## Data Availability

Data and materials are available upon request from the corresponding authors with the approval of all authors. The data are not publicly available due to privacy and ethical restrictions.

## Abbreviations

DHR	Drug Hypersensitivity Reaction
DPT	Drug provocation tests
NSAID	Nonsteroidal anti-inflammatory drug
OFC	Oral Food Challenge
PPI	Proton pump inhibitors
STAI	The State–Trait Anxiety Inventory
STAI-S	The State–Trait Anxiety Inventory – State
STAI-T	The State–Trait Anxiety Inventory – Trait
